# The Effects of Birth Order and Birth Interval on the Phenotypic Expression of Autism Spectrum Disorder

**DOI:** 10.1371/journal.pone.0051049

**Published:** 2012-11-30

**Authors:** Loren A. Martin, Narges L. Horriat

**Affiliations:** 1 Department of Graduate Psychology, Azusa Pacific University, Azusa, California, United States of America; 2 Department of Biology and Chemistry, Azusa Pacific University, Azusa, California, United States of America; The George Washington University, United States of America

## Abstract

A rise in the prevalence of diagnosed cases of autism spectrum disorder (ASD) has been reported in several studies in recent years. While this rise in ASD prevalence is at least partially related to increased awareness and broadened diagnostic criteria, the role of environmental factors cannot be ruled out, especially considering that the cause of most cases of ASD remains unknown. The study of families with multiple affected children can provide clues about ASD etiology. While the majority of research on ASD multiplex families has focused on identifying genetic anomalies that may underlie the disorder, the study of symptom severity across ASD birth order may provide evidence for environmental factors in ASD. We compared social and cognitive measures of behavior between over 300 first and second affected siblings within multiplex autism families obtained from the Autism Genetic Resource Exchange dataset. Measures included nonverbal IQ assessed with the Ravens Colored Progressive Matrices, verbal IQ assessed with the Peabody Picture Vocabulary Test, and autism severity assessed with the Social Responsiveness Scale (SRS), an instrument established as a quantitative measure of autism. The results indicated that females were more severely impacted by ASD than males, especially first affected siblings. When first and second affected siblings were compared, significant declines in nonverbal and verbal IQ scores were observed. In addition, SRS results demonstrated a significant increase in autism severity between first and second affected siblings consistent with an overall decline in function as indicated by the IQ data. These results remained significant after controlling for the age and sex of the siblings. Surprisingly, the SRS scores were found to only be significant when the age difference between siblings was less than 2 years. These results suggest that some cases of ASD are influenced by a dosage effect involving unknown epigenetic, environmental, and/or immunological factors.

## Introduction

Autism together with Asperger syndrome and pervasive developmental disorder - not otherwise specified (PDD-NOS) make up the classification of autism spectrum disorder (ASD). While a diagnosis of autism requires deficits in communication skills, atypical social interactions, and a restricted range of interests and/or repetitive motor movements before the age of 3, the disorders under the ASD umbrella have only the deficits in reciprocal social interactions in common [1]. Research on non-diagnosed siblings and parents of children with ASD has shown that they often exhibit some cognitive and behavioral deficits as well, thus classifying them within what is considered the broader autism spectrum [1], [2]. Based upon the study of a 2008 birth cohort, the prevalence of ASD is estimated to be 1 in 88, representing a 78% increase over a 2002 birth cohort [3]. Recurrence risk in siblings of ASD subjects is much higher, reported to be 21.7% when the broader autism spectrum was included in a recent study of 1235 families [4]. While autism has been shown to be largely hereditary [5], it is likely to have a multitude of causes given its behavioral diagnosis. Causes are likely to be diverse and include genetic, environmental and epigenetic factors [6], [7]. Indeed, cases of autism that are believed to be associated with a known genetic cause range between 10–20% but no single cause accounts for more than 1–2% of all ASD cases [8].

Given the high recurrence risk for ASD siblings, a few studies have focused on the behavioral phenotype of the affected siblings in multiplex families. In 1992, Lord published data from a small sample of 16 multiplex families that indicated a trend for decreasing nonverbal IQ scores with increasing birth order [9]. In 2001, Spiker et al. confirmed this earlier report by demonstrating a significant decline in nonverbal IQ scores between first affected and second affected siblings from 144 multiplex families [10]. While this sample was substantially larger than Lord’s original report, nonverbal IQ scores were also estimated from a variety of different tests based upon available diagnostic records. In addition, the broader phenotype was excluded from their analysis. Reichenberg et al. looked at birth order effects on ADI-R domain scores in 106 pairs of siblings with autism [11]. They found a significant decrease in useful phrase speech and a significant increase in repetitive behavior scores between first and second affected siblings. A breakdown of the repetitive behavior scores by domain categories indicated this significant difference was due to higher compulsion and circumscribed interests in the first affected siblings. Given that the first affected siblings were older at the time of testing, the influence of development may possibly explain the increase in repetitive behaviors [12]. Taken together, these studies demonstrate a pattern of decreasing IQ with increasing birth order in children affected with autism. It therefore appears that there is mounting evidence for an effect of birth order on autism symptom severity. However, all of these studies, while valuable for pointing towards a possible birth order affect in autism, were also limited by small sample sizes and the lack of systematic assessments.

The present study sought to examine the effects of autism birth order on measures of intelligence and autism symptoms in the largest dataset of multiplex families examined to date: the Autism Genetic Resource Exchange (AGRE) dataset. This dataset has been used extensively as a means to measure various trends in ASD causes and severity by analyzing the provided genotypic and phenotypic information. Our study specifically measured verbal and nonverbal IQ using the Peabody Picture Vocabulary Test (PPVT) and Ravens Colored Progressive Matrices (RCPM) respectively. In addition, autism symptoms were assessed including social skills by the Social Responsiveness Scales (SRS), and motor movements through the Repetitive Behavior Scales-Revised (RBS-R) and the Vineland Adaptive Behavior Scales (VABS). We also report for the first time the effects of birth order across affected siblings from an analysis of a limited number of families with at least 3 affected siblings.

## Methods

### Ethics Statement

Permission was granted to access the Autism Genetic Resource Exchange (AGRE; https://research.agre.org/) database through AGRE’s Scientific Steering Committee and an IRB exemption was granted through Azusa Pacific University’s Institutional Review Board (IRB# 12-09). Regulatory review, approval and oversight of AGRE’s human subject research were provided by Western IRB, Olympia, WA.

### Participants

The AGRE database is mostly comprised from families with more than one child diagnosed with ASD. Willing participant families complete a questionnaire and are registered with AGRE. A battery of tests and assessments are then administered, including the *Autism Diagnostic Interview-Revised* (ADI-R; [13]) in order to confirm the diagnosis. The AGRE data utilizes three categories of designation for ASD diagnosis: Autism, Not Quite Autism (NQA), and Broad Spectrum. NQA designates individuals who are no more than one point away from meeting full autism criteria on any or all of the three "content" domains (i.e. social, communication, and/or repetitive behavior) of the ADI-R, and meet criteria on the “age of onset” domain; or, individuals who meet criteria on all three "content" domains, but do not meet criteria on the "age of onset" domain. Since the ADI-R does not provide validated algorithms to designate the broader autism spectrum, cases of PDD-NOS and Asperger’s’ Disorder are determined using a combination of the ADI-R and other diagnostic instruments such as the Autism Diagnostic Observation Schedule (ADOS) [14].

### Procedure

Our analyses of the AGRE phenotype data focused on the results of the following measures: Peabody Picture Vocabulary Test (PPVT-III; [15]), Ravens Colored Progressive Matrices (RCPM; [16]), Vineland Adaptive Behavior Scales (VABS; [17], Repetitive Behavior Scale- Revised (RBS-R; [18]) and Social Responsiveness Scale (SRS; [19]). The sample size for each measure varied as the following criteria was used to determine each sample: 1) Data had to be available from both the first and second sibling diagnosed with autism, NQA, or the broader ASD spectrum within each multiplex family. 2) Both the first and second affected siblings each had to be from a single birth in order to avoid potential confounds associated with twinning/multiple births. 3) The ASD diagnosis for both the first and second affected siblings had to be independent of any indicated comorbid disorder (e.g. fragile X syndrome). 4) The first and second affected siblings had to share the same biological mother. It should be noted that we chose to use the term “affected” rather than “born” in recognition of the fact that approximately 1/3 of ASD cases involve a period of normal development followed by regression [20]–[22]); thus, it is yet unclear whether all children with ASD are born with the disorder. In this study, we focused on affected order and not birth order in general. Nevertheless, in all cases affected order corresponded with birth order.

Data for the selected instruments, along with the AGRE pedigree file, were downloaded as Microsoft Excel spreadsheets. The data were sorted by family ID and then by age. Actual birth order and autism birth order were then determined and assigned based upon the age of each proband on the date of testing. Data were then selected for analyses based upon the criteria described above.

### Instrumentation

The AGRE database includes data from a number of instruments measuring various aspects of functioning; however, we specifically focused on measures that would reveal information on intelligence (verbal and nonverbal), adaptive behaviors, motor functions, and social behavior.

#### Peabody picture vocabulary test (Peabody or PPVT)

The PPVT-III is an individually administered, norm-referenced assessment of receptive vocabulary. It measures verbal IQ by testing the participant’s ability to point to the correct picture out of four that matches a spoken vocabulary word. Apart from a full intelligence test, the PPVT-III is the most widely used measure to test for verbal competence within similar studies of ASD [23].

#### Ravens colored progressive matrices (Ravens or RCPM)

The Ravens is an individually administered, norm-referenced assessment of nonverbal processing, including the ability to discern perceptual relations and reason by analogy, that requires the participant to identify the missing piece in a visual pattern [16]. Similar to the PPVT-III for measuring verbal IQ, the Ravens is the most widely used measure for the assessment of nonverbal cognitive ability in ASD studies [23].

#### Vineland adaptive behavior scales (VABS)

The VABS is an assessment based on parental rating of their child’s age-appropriate, socially adaptive behaviors [17]. This widely used and respected measure demonstrates strong reliability and validity for children exhibiting typical and atypical patterns of development. The VABS provides standard scores (M = 100, SD = 15) for four skill domains: communication, daily-living, socialization, and motor. Because these domains closely mirror the areas of delayed/abnormal development in ASD, it is an excellent measure for the assessment of adaptive functioning in children with autism. It should be noted that for this particular instrument as with the Peabody and the Ravens, higher scores indicate better functioning.

#### Repetitive behavior scale-revised (RBS-R)

The RBS-R is an expanded version of the RBS that was designed to measure the broad range of repetitive behavior observed in autism [18]. It is an informant-based, 43 item questionnaire, with established reliability and validity [24]. Respondents are asked to rate a child’s behavior observed over the past month on a 4-point Likert scale. As opposed to the above measures, higher scores indicate more severe repetitive behaviors.

#### Social responsiveness scale (SRS)

The SRS measures the severity of social impairment in ASD. It is an instrument based on parent and teacher rating scales of the child’s social behaviors with established reliability and validity [19]. This particular measure focuses on identifying the presence and extent of social impairment. It determines the severity of social impairment by analyzing behaviors on a quantitative scale. Similar to the RBS-R, a high score indicates more severe symptoms of autism.

### Data Analysis

Data were analyzed using IBM SPSS statistics version 18. Comparisons between first and second affected sibling sets were carried out using paired sample t-tests. Relationships between the behavioral data and potential confounds were first explored using Pearson’s R and when deemed necessary the effects of these potential confounds were controlled for using ANCOVA. A comparison across first, second, and third affected siblings was conducted on the RCPM data using a repeated measures ANOVA. Frequency analyses were also conducted using Chi-square.

## Results

### Significant Effects of Birth order on Verbal and Nonverbal IQ

**Figure 1:**
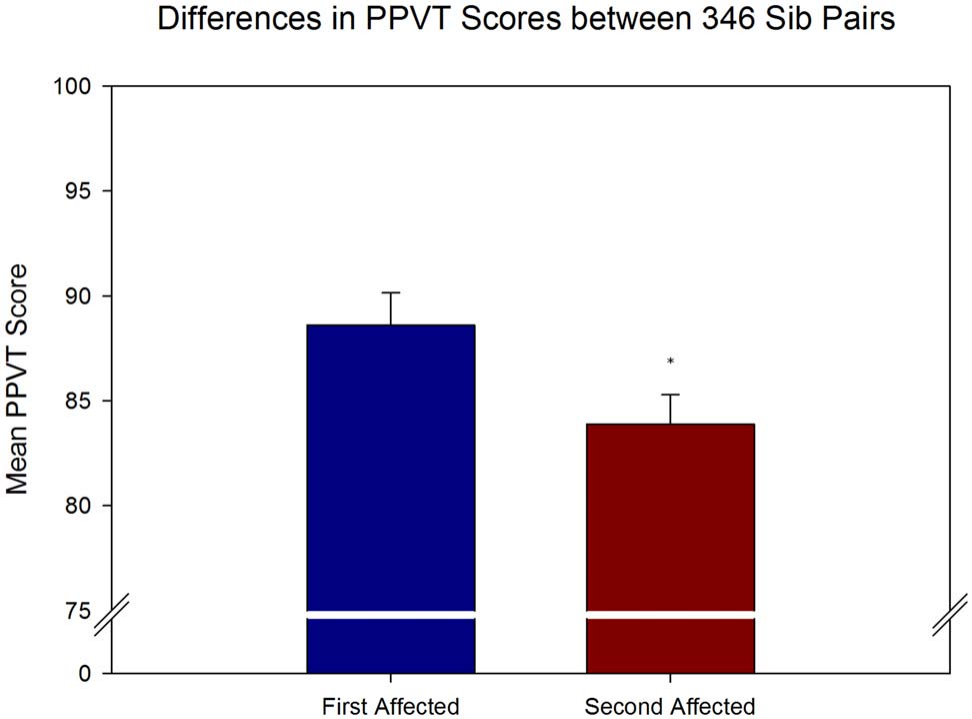
Mean PPVT scores across first and second affected siblings. Paired-samples t-tests revealed a significant difference in the mean scores on the PPVT across first and second affected siblings (t(345) = 2.997, p = .003). Results indicate a significant decline in verbal IQ across birth order. Error bars represent SEM and asterisk indicates significant difference.

**Figure 2:**
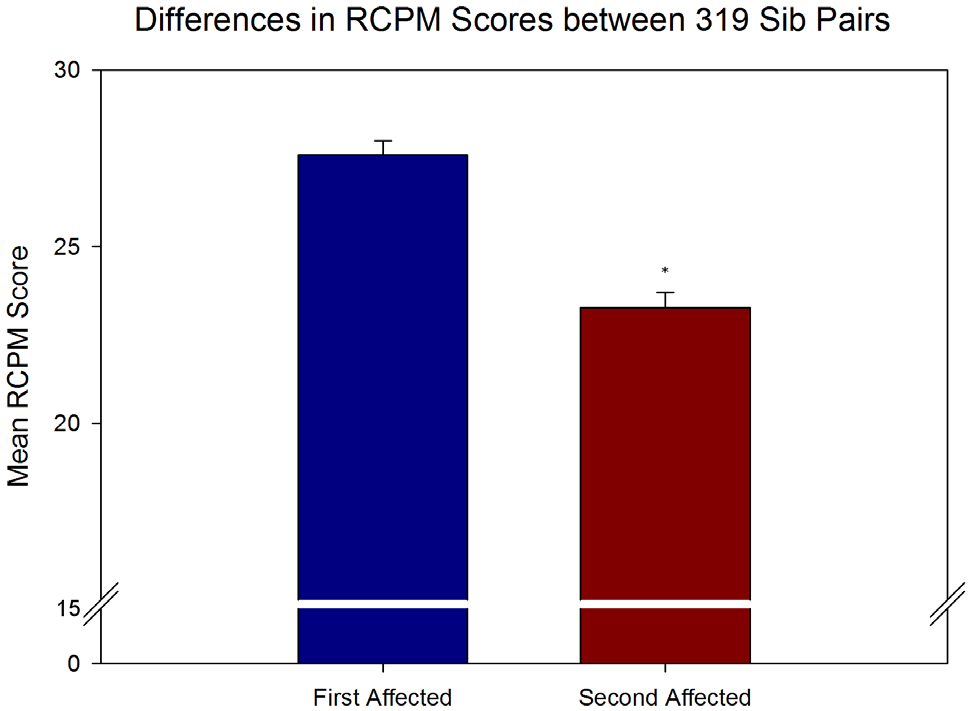
Mean scores on the RCPM across first and second affected siblings. Paired-samples t-tests showed significant differences in the mean scores on the RCPM across first and second affected siblings (t(318) = 10.45, p<.001). Results indicate a significant decline in nonverbal IQ across birth order. Error bars represent SEM and asterisk indicates significant difference.

Verbal and nonverbal IQ scores were compared between first affected and second affected siblings in multiplex autism families using paired-samples t-tests. Results from the analysis of PPVT scores from 346 sib pairs demonstrated significantly higher verbal IQ scores (t(345) = 2.997, p = .003) for the first affected siblings. As shown in Figure 1, the mean verbal IQ score for the first affected siblings was 88.60 (SD = 28.80) and for the second affected siblings was 83.88 (SD = 26.05). For the RCPM, data from 319 sib pairs demonstrated significantly higher nonverbal IQ scores (t(318) = 10.45, p<.001) for the first affected siblings. The mean score from the RCPM for the first affected siblings was 27.60 (SD = 6.93) and for the second affected siblings was 23.27 (SD = 7.73) as shown in Figure 2. In doing our analysis of both the PPVT and RCPM, we noticed a disproportionate number of second affected siblings (39.4% and 41.8%, respectively) were deemed untestable compared to first affected siblings (19.6% and 21.5%, respectively; see Table 1). Chi-squared analyses (with Yates correction) confirmed there was a significant association between affected order and testability (PPVT χ^2^(1) = 63.36, p<.0001; RCPM χ^2^(1) = 61.22, p<.0001). Based upon the odds ratio, second affected siblings were 2.68 times more likely to be deemed untestable than first affected siblings for the PPVT and 2.58 times more likely to be deemed untestable for the RCPM. It is worth noting that in our limited sample of third affected siblings for both the PPVT and RCPM (n = 80 for each test), 41.3% of the PPVT sample and 47.5% of the RCPM sample were deemed untestable. The reason given for the majority of untestable cases was because the child was too low functioning.

**Table 1:** Percentage of siblings deemed untestable in the PPVT and RCPM.

Assessment	First Affected	Second Affected	Third Affected
**PPVT**	19.6%	39.4%	41.3%
**RCPM**	21.5%	41.8%	47.5%

Note: Most cases were deemed untestable due to the child being too low functioning.

### Effects of Age and Sex on IQ Measures

Because most of the sib pairs were assessed on the same date, the first affected siblings were older than the second affected siblings at assessment (see Table 2). In order to determine if age influenced the results for the PPVT and RCPM described above, Pearson correlations were carried out between age and test scores for both first and second affected siblings. For the PPVT, there was no correlation between age and test score for the first affected siblings (r = −.009, p = .864) and a weak negative correlation between age and test score for second affected siblings (r = −.161, p = .003). This weak negative relationship was opposite to our findings that first affected siblings, and thus eldest siblings, had higher PPVT scores than second affected siblings and therefore was not responsible for the significant effects of autism birth order on PPVT scores. For the RCPM, there were significant positive correlations between age and test score for both the first (r = .385, p<.001) and second (r = .540, p<.001) affected siblings. It was therefore, necessary to control for the influence of age on autism birth order. ANCOVA revealed that the covariate, age, was significantly related to the test scores (F(1, 637) = 167.99, p<.001). However, there was also a significant effect of affected order after controlling for the effect of age (F(1, 637) = 8.32, p = .004).

**Table 2:** Demographic information including sex and mean age at assessment.

Assessment	Total # ofSib-pairs	# of Females		# of Males		Mean Age (SD)	
		FirstAffecteds	SecondAffecteds	FirstAffecteds	SecondAffecteds	FirstAffecteds	SecondAffecteds
**PPVT**	346	76	85	270	261	10.87 (4.1)	7.80 (3.5)
**RCPM**	319	71	83	248	236	11.01 (3.9)	8.07 (3.3)
**VABS**	515	104	123	411	392	11.08 (4.8)	8.18 (4.3)
**RBS**	98	32	25	66	73	11.35 (4.5)	8.37 (4.2)
**Parent SRS**	380	83	93	297	287	10.90 (4.7)	8.10 (4.3)
**Teacher SRS**	107	21	26	86	81	12.81 (4.9)	9.89 (4.1)

Out of the 346 sib pairs in the PPVT dataset, there were 270 male and 76 female first affected siblings and 261 male and 85 female second affected siblings. An independent-samples t-test demonstrated significantly higher PPVT scores for males versus females in the group of first affected siblings (t(344) = 2.409, p = .017), but not for the second affected siblings (t(344) = −1.066, p = .287). Despite the effect of sex on PPVT scores in the group of first affected siblings, an analysis of the subgroup of 214 sib pairs comprised of only males demonstrated that the effect of autism birth order on PPVT scores is still highly significant (t(213) = 3.370, p = .001). Out of the 319 sib pairs in the RCPM dataset, there were 248 male and 71 female first affected siblings and 236 male and 83 female second affected siblings. Similar to the PPVT scores, independent-samples t-tests revealed significantly higher RCPM scores for males versus females in the group of first affected siblings only (first affected: t(317) = 2.658, p = .009; second affected: t(317) = 1.378, p = .169). As with the PPVT scores though, an analysis of the subgroup of 193 male-male sib pairs showed that the effect of autism birth order on RCPM scores was still highly significant (t(192) = 8.428, p<.001). For both tests, the number of female-female sib pairs was too small for similar comparisons.

### Effects of Birth Order on Motor Behavior

Comparisons were also made between 515 affected sib pairs on the VABS and its major domains using paired-samples t-tests. There were no significant differences in the composite scores between the sib pairs (t(514) = −.045, p = .964). For the major domains of the VABS including Communication, Daily Living Skills, Social Skills, and Motor Skills, only the Motor Skills domain was significantly different (t(514) = 3.82, p<.001). The first affected siblings had a higher Motor Skills score (M = 78.49, SD = 31.65) than the second affected siblings (M = 73.73, SD = 25.57). However, Pearson correlations between age and Motor Skills scores revealed a significant positive correlation for first affected siblings (r = .113, p = .010) but not for second affected siblings (r = .068, p = .121). After controlling for age using ANCOVA, the Motor Skills domain was no longer significantly different between sib pairs (F(1,1027) = 2.631, p = .105).

Data was available for only a limited number of sib pairs (n = 98) from the Repetitive Behavior Scales. While the mean scores indicated a trend for lower repetitive behavior in first affected siblings (M = 26.59, SD = 20.65) compared to second affected siblings (M = 29.28, SD = 21.22), the paired-samples t-test did not yield significant results (t(97) = −1.48, p = .143).

### Significant Effects of Birth Order on Autism Severity

**Figure 3:**
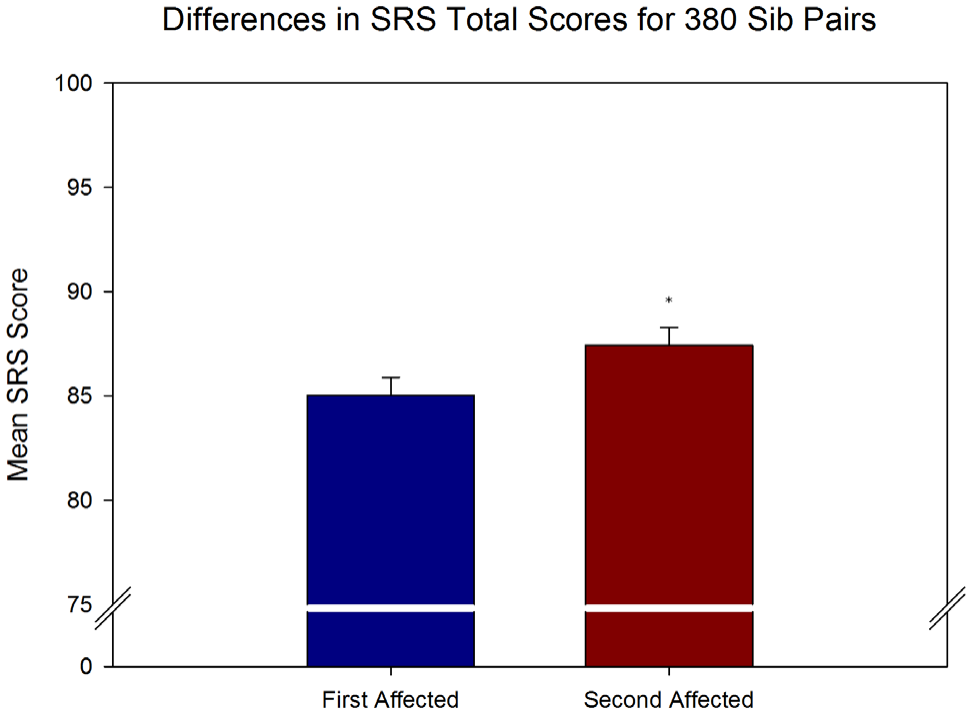
Mean scores on the SRS across first and second affected siblings. Paired-samples t-tests of SRS data demonstrated significant increase in autism severity between first and second affected siblings (t(379) = −2.250, p = .025). These results are consistent with an overall decline in function as indicated by the IQ data. Error bars represent SEM and asterisk indicates significant difference.

The SRS is largely considered the best quantitative measure of autistic symptoms. Therefore, in order to compare overall degrees of autism between first and second affected siblings in multiplex autism families, we conducted a paired-samples t-test on the SRS scores from parent interviews of 380 sib pairs. Results showed that first affected siblings had significantly lower SRS scores (M = 85.03, SD = 16.57) than second affected siblings (M = 87.43, SD = 16.57) indicating a slightly higher degree of autism in the second affected siblings (t(379) = −2.250, p = .025; Figure 3). Frequency analysis demonstrated that 210 out of 380 sib pairs demonstrated this pattern with another 11 sib pairs having identical SRS scores. After removing the 11 sib pairs with identical scores, chi-square analysis confirmed that a significantly greater proportion of sib pairs (56.9%) demonstrated a pattern of higher SRS scores in the second affected siblings (χ^2^ = 7.049, p = .008). A much smaller sample of SRS scores was available from teacher interviews (n = 107 sib pairs). A paired-samples t-test indicated a trend for lower SRS scores from first affected siblings (M = 67.61, SD = 11.51) compared to second affected siblings (M = 70.26, SD = 10.65) consistent with the parent interview data (t(106) = −1.812, p = .073).

### Effects of Age and Sex on SRS Scores

As with the other measures, most of the sib pairs were assessed on the same date and thus the first affected siblings were older than the second affected siblings at assessment (see Table 2). A Pearson correlation between age and SRS scores was significant for first affected siblings (r = .120, p = .019) but not for second affected siblings (r = .058, p = .262). In order to control for the effect of age on the SRS findings, we conducted an ANCOVA with age at assessment as the covariate. Results from the SRS ANCOVA demonstrated a significant effect of the covariate, age at assessment, on the SRS scores (F(1, 757) = 6.226, p = .013). However, there was also a significant effect of autism birth order after controlling for the age at assessment (F(1, 757) = 7.092, p = .008).

In order to ensure that our significant results were not driven by the sex of the siblings, we controlled for the effects of both age and sex using ANCOVA. Results demonstrated that the SRS scores were still significantly higher for second affected siblings after controlling for both of these factors (F(1, 756) = 6.610, p = .010). However, a separate analysis using a paired-samples t-test revealed no significant difference in total SRS scores between first and second affected male only sib pairs (t(230) = −1.636, p = .103).

### Effects of Birth Order on Autism Severity Specific to Sib Pairs Born within 2 Years

**Figure 4:**
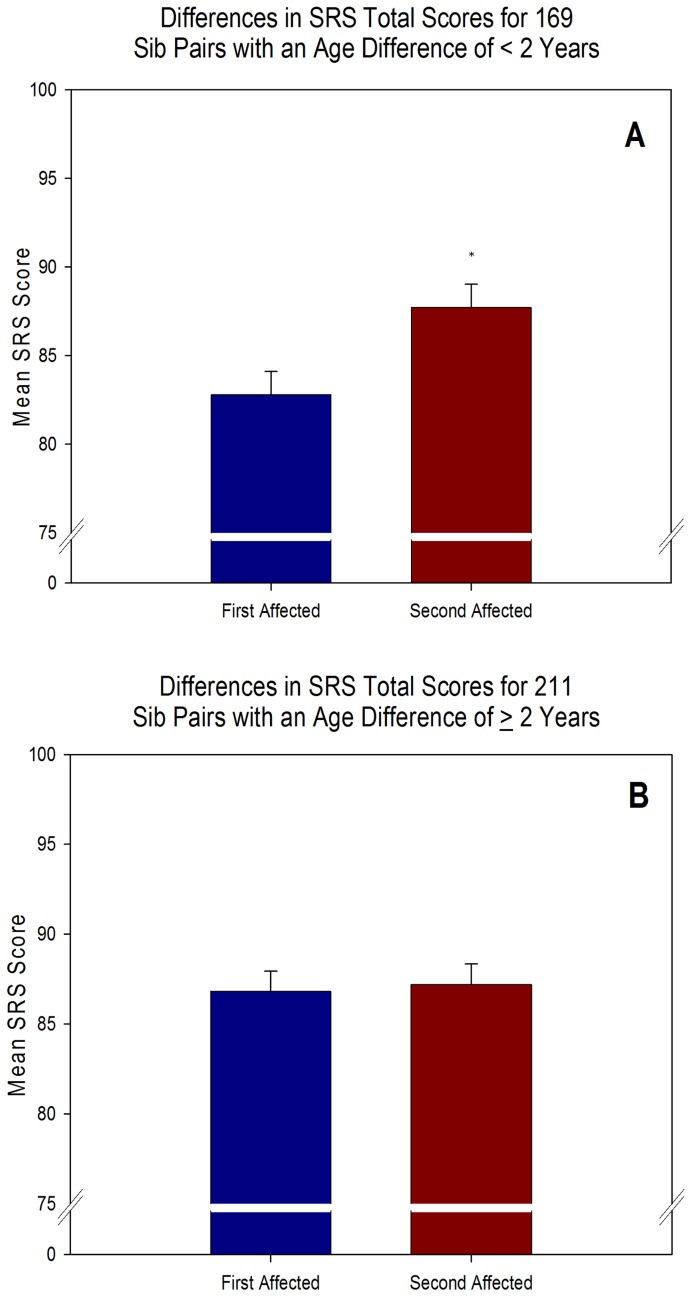
SRS data comparisons based upon the age difference of the sib pairs. A) When the age gap between the ASD sib pairs was less than 2 years, the differences between the total SRS scores were highly significant (t(168) = −3.232, p = .001). B) When the age gap between ASD sib pairs was greater than or equal to 2 years, the total SRS scores were nearly equivalent (t(210) = −266, p = .791). Error bars represent SEM and asterisk indicates significant difference.

We found a surprising effect of the age difference between sib pairs on our results. For most of the datasets, the age at the time of testing was only given in whole years. However, for the SRS dataset, the age at the time of testing included fractions of years allowing for a more detailed analysis of birth interval. We separately analyzed all SRS parent interview scores from sib pairs born within 2 years of each other (n = 169) and all sib pairs with an age difference greater than or equal to 2 years (n = 211). Paired-samples t-tests indicated that autism birth order had a highly significant effect on SRS scores in sib pairs with an age difference less than to 2 years (t(168) = −3.232, p = .001). Consistent with our overall analysis, first affected siblings (M = 82.80, SD = 16.93) had significantly lower scores than second affected siblings (M = 87.72, SD = 16.90; see Figure 4A). Chi-square analysis demonstrated that a significantly greater proportion of sib pairs (61.5%) had a pattern of higher SRS scores in the second affected siblings (χ^2^ = 8.503, p = .004; 8 sib pairs with identical scores excluded). On the other hand, paired-samples t-tests indicated that autism birth order did not affect SRS scores in sib pairs with an age difference greater than or equal to 2 years (t(210) = −.266, p = .791). In fact, the mean scores of the first (M = 86.82, SD = 16.09) and second (M = 87.21, SD = 16.34) affected siblings were nearly equivalent (see Figure 4B). This same pattern was found in the SRS scores from teacher interviews. In sib pairs with an age difference of less than 2 years, first affected siblings (M = 65.50, SD = 10.23) had significantly lower SRS scores than second affected siblings (M = 69.85, SD = 10.56; t(45) = −2.138, p = .038) but in sib pairs with an age difference greater than or equal to 2 years, there was no difference between SRS scores of first (M = 69.20, SD = 12.22) and second (M = 70.57, SD = 10.79) affected sib pairs (t(60) = −.668, p = .506).

In order to determine if the differences in the SRS scores between first and second affected siblings in the group of sib pairs with an age difference less than to 2 years was driven by a particular domain of the SRS we conducted paired-samples t-tests on scores for each of the 5 domains of the SRS. Results demonstrated that the scores for each SRS domain were significantly lower for first affected siblings than second affected siblings (see Table 3).

**Table 3:** SRS domain scores and t-test results for 169 sib pairs born within 2 years of each other.

SRS Domain	Mean of first affectedsiblings (SD)	Mean of second affectedsiblings (SD)	t score	p value
Awareness	71.91 (14.6)	76.54 (16.2)	−3.288	.001
Cognition	78.62 (15.3)	83.21 (14.8)	−3.631	<.001
Communication	79.79 (16.2)	84.68 (15.4)	−3.370	.001
Motivation	72.59 (15.7)	75.86 (15.0)	−2.099	.037
Mannerism	85.87 (19.4)	89.29 (20.1)	−2.017	.045

A Pearson correlation between the age difference of the sib-pairs and the SRS score difference between the sib pairs indicated that the birth order effect of the sib pairs born within 2 years of each other was not related to the age gap (r = .043, p = .582). Furthermore, independent Pearson correlations between the age at testing and SRS scores for both first and second affected siblings indicated that age was not related to SRS score (first affecteds: r = .052, p = .504; second affecteds: r = −.035, p = .655). Thus, the effect of birth order on SRS scores was not driven by the age difference between siblings.

Despite the significant differences in SRS scores between first and second affected siblings, these scores were still significantly correlated (r = .321, p<.001), likely reflecting the high heritability previously shown for SRS scores [25], [26]. However, the correlation of SRS scores across sib pairs with an age gap greater than 2 years was not significant (r = .120, p = .081).

The breakdown by sex of first and second affected siblings was similar in the group of 169 sib pairs with an age difference less than 2 years. There were 31 females and 138 males in the first affected siblings and 42 females and 127 males in the second affected siblings. The breakdown by sex of first and second affected siblings was nearly identical in the group of 211 sib pairs with an age difference greater than 2 years (first affecteds = 52 females, 159 males and second affecteds = 51 females, 160 males). In the group of sib pairs with an age difference less than 2 years, there were 106 male pairings, 10 female pairings, 21 female-male pairings, and 32 male-female pairings. In the group of sib pairs with an age difference greater than or equal to 2 years, there were 125 male pairings, 17 female pairings, 35 female-male pairings, and 34 male-female pairings. Independent-samples t-tests showed that there was an overall effect of sex on SRS scores for both first (t(378) = −2.633, p = .009) and second (t(378) = −2.393, p = .018) affected siblings with males scoring significantly lower than females in both categories. However, the scores of the first affected siblings were still significantly lower than the scores of the second affected siblings in the group of sib pairs with an age difference less than 2 years when only the subgroup of 106 male-male sib pairs were analyzed (t(105) = −2.130, p = .036). In the subgroup of 125 male-male pairings of the group of sib pairs with an age difference greater than or equal to 2 years, there was still no difference between first and second affected siblings (t(124) = −.338, p = .736).

### Analysis of Sib Triads

**Figure 5:**
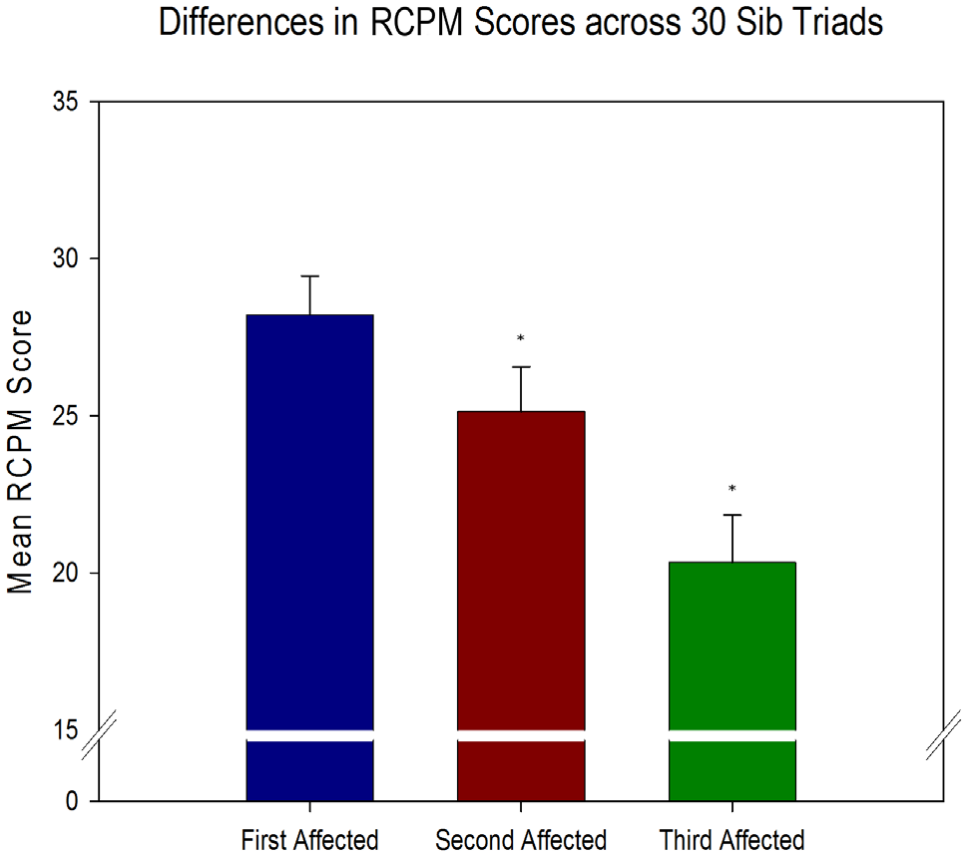
Mean scores on the RCPM across first, second, and third affected siblings. Although comparisons of sib triads were not possible on most assessments due to the small sample size, an analysis of mean RCPM scores from 30 sib triads using a repeated measures ANOVA revealed a significant decline in nonverbal IQ across affected siblings (F(2,58) = 8.14, p = .001). Error bars represent SEM and asterisk indicates significant difference.

For a limited number of multiplex autism families, data was available from at least 3 affected siblings. For most of the measures, the n was too small for adequate statistical power. However, even with only 30 sib-triads, the RCPM was significantly different across birth order (F(2,58) = 8.14, p = .001) with a mean of 28.2 for the first affected siblings, 25.1 for the second affected siblings, and 20.3 for the third affected siblings (Figure 5).

## Discussion

The Autism Genetic Resource Exchange was established in 1997 by Cure Autism Now (CAN) to provide genotypic and phenotypic information for researchers. The database has primarily been used for genetic studies on autism and the broader spectrum disorders. In this study, we compared data between over 300 sib pairs diagnosed with ASD or the broader autism spectrum. Results demonstrate that the second affected sibling is on average more severely impaired than the first affected sibling. This is evident by reduced verbal and nonverbal IQ scores in second affected siblings and increased SRS scores, an index of autism severity. It is also supported by lower motor skills scores and a trend for more repetitive behaviors among second affected siblings as measured through the VABS and RBS-R, respectively. While the differences in each measure represent less than a single standard deviation, they are nonetheless significant in all measures with the exception of the RBS-R which was limited by a much smaller sample size. In addition, they represent a reliable pattern across behavioral measures and are consistent with previous studies using smaller datasets [9], [10]. For a summary of the results from all of these assessments see Table 4.

**Table 4:** Summary of the results from all assessments.

Test	Measure	n	Mean of first affectedsibling(SD)	Mean of secondaffectedsibling (SD)	t score	p value	Overall Sig.	Sig. after controlling age and sex
PPVT	Verbal IQ	346	88.60 (28.80)	83.88 (26.05)	2.997	0.003	Yes*	Yes
RCPM	Nonverbal IQ	319	27.60 (6.93)	23.27 (7.73)	10.45	<0.001	Yes*	Yes
VABS	Motor Skills	515	78.49 (31.65)	73.73 (25.57)	3.82	<0.001	Yes	No
RBS	Repetitive Behavior	98	26.59 (20.65)	29.28 (21.22)	−1.48	0.143	No	No
SRS	Social Skills	380	85.03 (16.57)	87.43 (16.57)	−2.250	0.025	Yes**	Yes

* In addition to these results, second affected siblings were 2.68 times more likely to be deemed untestable than first affected siblings for the PPVT and 2.58 times more likely to be deemed untestable for the RCPM.

** Further analysis revealed SRS results were driven by differences between affected pairs born <2 years apart.

In addition to the differences in ASD severity that we found between affected siblings, we also found sex differences in symptom severity. First affected females had lower verbal and nonverbal IQ scores than first affected males, and both first and second affected females had higher SRS scores than the males. These results are consistent with previous studies that have reported lower IQ in females diagnosed with ASD [27]–[29]. It is interesting to note that in one of these studies, Banach et al. found females to have lower IQ than males in a sample of simplex families but not in a sample of multiplex families. However, the authors randomly chose the affected female and male probands for their comparisons in the multiplex sample. Given the interaction effects of the affected order on the IQ sex differences observed in our study, it is possible that similar results would have been obtained in the Banach et al. study if the affected probands had not been randomly selected for comparison.

The differences in ASD severity that we have observed between affected siblings resonate well with two separate studies published in the past year that explored the occurrence risk of ASD rather than the severity of the disorder. In the first study, Cheslack-Postava et al. found that the odds of an autism diagnosis were significantly higher in second-born children relative to their first-born siblings when the interpregnancy interval (IPI) was less than 24 months [30]. This finding is remarkable in light of the significant relationship between the age difference of affected siblings and ASD severity as indicated by the SRS in our study. In the most recent study, Turner et al. reported significantly more diagnoses of ASD in second-born siblings than first-born siblings [31]. This pattern was observed in a dataset of simplex families as well as two multiplex datasets including the AGRE dataset used for our analyses. These findings are in accord with our own results indicating increased ASD severity for second affected siblings and likely stems from a common etiological factor. For example, it is possible that an ASD causal factor manifests subclinically in some first-borns while resulting in a mild, yet diagnosable form of ASD in other first-borns.

Our findings of increased autism severity across birth order also resonate with several studies that have demonstrated a significant relationship between parental age and ASD diagnosis [32], [33]. Parents are of course older when the later-born siblings are conceived than they were at the conception of their earlier born counterparts. It is quite possible that the data supporting parental age as a risk factor for ASD is simply a reflection of this birth order effect.

Through a chi-square analysis, the pattern of increased autism severity across sib pairs was found in a significantly greater proportion of multiplex families than expected (56.9%). However, 43.1% of multiplex families demonstrated the reverse pattern. This is most likely due to the multi-causal nature of ASD and the genetic variance between affected siblings. It is likely that many causes of ASD when combined with the genetic variance across affected sib pairs result in a random manifestation of symptoms that are equally distributed between affected siblings. However, this data indicates that at least one factor in the cause of ASD symptoms manifests itself disproportionately across sib pairs so that the second affected sibling is more severely impacted. This suggests a dose-response relationship for an ASD causal factor(s). There are a few putative causes of autism that may act through a dose-response relationship with the developing brain. In addition, the impact of the social environment must be considered.

### Methylation of the Oxytocin Receptor Gene

A putative role for the neurohormone oxytocin in the etiology of autism was postulated long ago due to the importance of oxytocin (OT) in prosocial behaviors [34], [35]. An early study linking OT with autism demonstrated lower plasma levels of OT and correlations between plasma OT levels and social functioning [36]. More recent studies have found positive linkage between the 3p25 chromosomal region containing the oxytocin receptor (OXTR) gene and autism [37]–[39]. Other studies have found associations between ASD and two single nucleotide polymorphisms (SNPs) within the OXTR gene [40]–[42]. In addition to the mounting evidence of a connection between autism and the OT system, a few recent studies have demonstrated therapeutic value in the delivery of exogenous OT to individuals with ASD including reduced repetitive behavior and increased social behavior [43]–[46].

Perhaps the most exciting finding to date related to the role of the OT system in the etiology of autism was the recent discovery of significant increases in DNA methylation in the promoter region of the OXTR gene in blood and brain samples from autistic individuals compared to controls [47]. DNA methylation is a known epigenetic mechanism of gene silencing and in this same study, increased methylation was associated with decreased OXTR gene expression in the temporal cortex of autism cases.

DNA methylation has been shown to be influenced by environmental factors in both honey bees and rodents. In the honey bee, genetically identical larvae will either become fertile queens or sterile workers dependent upon whether they are fed royal jelly produced by nurse bees. Researchers have shown that the interruption of DNA methylation at a precise moment in development leads to the fertile queen phenotype and therefore the royal jelly is presumed to halt DNA methylation [48]. In mice carrying a dominant mutation at the agouti gene, DNA methylation controls phenotypes of coat color and obesity. Manipulation of the maternal diet before, during and after pregnancy has been shown to influence DNA methylation of this gene as well as others [49], [50]. Recent studies in both mice and rats have shown that paternal diet can also influence DNA methylation and alter gene expression in offspring [51], [52]. Finally, maternal nurturing behaviors have been shown to influence the methylation status of the glucocorticoid receptor promoter in the hippocampus of rat pups [53].

The burgeoning evidence of environmental influences on DNA methylation opens up the possibility that methylation of the OXTR gene is similarly influenced by environmental mechanisms. It is conceivable that a methylation promoting agent accumulates in a parent over time leading to increased silencing of the OXTR gene across susceptible offspring and therefore resulting in increased severity across autism birth order. OXTR is just one of several genes that have been linked to autism etiology. The expression of several autism susceptibility genes may be controlled by epigenetic mechanisms.

### Maternal Immune Response During Pregnancy

Immunological factors have long been implicated in the etiology of autism (for review see [54]). However, the first suggestion that autism may be caused by a maternal immune response during pregnancy came from a study by Warren et al. in which 6 of 11 mothers of children with autism were reported to carry antibodies reactive to lymphocytes from their autistic child [55]. Since this early study, several recent reports have demonstrated the presence of maternal antibodies against fetal brain tissue in mothers of children with autism [56]–[59]. In addition, both mice and monkeys exposed to antibodies from mothers of children with autism have demonstrated abnormal behavior suggesting a pathogenic nature for these antibodies [60]–[62].

There are at least two possible models to explain the maternal immune response. The first model involves maternal antibodies raised against a foreign pathogen and then cross-reacting with a fetal brain protein. In the second model, pathogenic maternal antibodies target the fetal brain protein directly. Both of these possibilities may offer an explanation for the present data demonstrating that symptom severity increases with autism birth order within a family. Also, in either model, it is possible that unaffected children within multiplex families express a non-reactive isoform of the antibody-reactive protein. However, the second model seems to be the best explanation for the present data. In this model, the mother would have generated antibodies against the fetal brain protein during her pregnancy with the first affected child. For her pregnancy with the second affected child though, the mother would already be carrying pathogenic antibodies from the first pregnancy plus generating additional antibodies leading to a higher titer of pathogenic antibodies. This would especially be true for pregnancies that are close together as the antibody titer would decrease with time. Thus, this model not only fits our data indicating a propensity for increased severity across autism birth order, but also our data from SRS scores suggesting this effect is specific to siblings born within 2 years of each other.

### Social Environmental Factors

The differences in autism severity between sib pairs may potentially be due to socio–environmental factors. For example, it is conceivable that second affected siblings received less parental attention early in development due to divided care between multiple offspring. Indeed, birth order data from typically developing children indicates that first born children do enjoy a period of parental attention that is not divided between other children [63]. Furthermore, children with parents who are highly involved exhibit fewer social and behavioral problems [64]. This seems to validate the idea that first born children inadvertently are more behaviorally well-adjusted simply due to the “undivided” attention they receive. However, in the case of our study, this socio-environmental effect is confounded by the fact that the second affected siblings are diagnosed at an earlier age than first affected siblings due to greater autism awareness of the caregiver [65]. This earlier diagnosis would presumably result in earlier intervention for the second affected siblings. Several studies have shown that early intervention results in better outcomes in ASD [66]–[69]. Therefore, it would be predicted that the autism phenotype would be partially rescued to a greater degree in second affected siblings than first affected siblings because of earlier diagnosis and intervention.

The SRS data alone appears to support to the synthesis of these potentially competing influences on the autistic phenotype. Given that the score differences were only significant for sib pairs with an age difference of less than 2 years, it is possible that these differences were driven by the demands of caring for two children under the age of 2. As the age difference between siblings extends beyond this threshold, the caregivers would presumably be able to devote more attention to the younger sibling. However, this theory does not explain why there is an increased risk of ASD in second born siblings when IPI is less than 2 years [30]. Thus, when our results are considered together with the results from the Cheslack-Postava et al. study, social environmental factors seem highly unlikely.

Overall, accumulated evidence from our study and others seems to point to at least one ASD causal factor that operates in a dosage-like fashion. With minimal exposure to the developing fetus, this unknown causal agent results in either subclinical symptoms in the case of non-diagnosed first-born siblings or a more mild form of ASD. In subsequent pregnancies, there exists a greater chance that this unknown causal agent will have accumulated resulting in more cases of ASD in second-born siblings as well as more severe ASD symptoms. With an increase in the amount of time between pregnancies/births, the mother’s body would have more time to adjust to or eliminate this causal factor leading to greater protection to the developing fetus. The increased IPI could thereby prevent more ASD diagnoses and more severe ASD impairments. Future studies on ASD causal factors may benefit by focusing on families exhibiting a pattern of increased ASD severity in second affected siblings born within 2 years of their less severely affected first-born sibling.

## References

[1] Dawson G, Estes A, Munson J, Schellenberg G, Bernier R, et al (2007) Quantitative assessment of autism symptom-related traits in probands and parents: Broader Phenotype Autism Symptom Scale. J Autism Dev Disord 37: 523–536. 16868845 10.1007/s10803-006-0182-2

[2] Bishop DV, Maybery M, Maley A, Wong D, Hill W, et al (2004) Using self-report to identify the broad phenotype in parents of children with autistic spectrum disorders: a study using the Autism-Spectrum Quotient. J Child Psychol Psychiatry 45: 1431–1436. 15482503 10.1111/j.1469-7610.2004.00849.x

[3] Prevalence of autism spectrum disorders–Autism and Developmental Disabilities Monitoring Network, 14 sites, United States, 2008. MMWR Surveill Summ 61: 1–19. 22456193

[4] Constantino JN, Zhang Y, Frazier T, Abbacchi AM, Law P (2010) Sibling recurrence and the genetic epidemiology of autism. Am J Psychiatry 167: 1349–1356. 20889652 10.1176/appi.ajp.2010.09101470 PMC2970737

[5] Bailey A, Le Couteur A, Gottesman I, Bolton P, Simonoff E, et al (1995) Autism as a strongly genetic disorder: evidence from a British twin study. Psychol Med 25: 63–77. 7792363 10.1017/s0033291700028099

[6] Szatmari P, Jones MB, Zwaigenbaum L, MacLean JE (1998) Genetics of autism: overview and new directions. J Autism Dev Disord 28: 351–368. 9813773 10.1023/a:1026096203946

[7] Szatmari P (1999) Heterogeneity and the genetics of autism. J Psychiatry Neurosci 24: 159–165. 10212560 PMC1188998

[8] Abrahams BS, Geschwind DH (2008) Advances in autism genetics: on the threshold of a new neurobiology. Nat Rev Genet 9: 341–355. 18414403 10.1038/nrg2346 PMC2756414

[9] Lord C (1992) Birth order effects on nonverbal IQ in families with multiple incidence of autism or pervasive developmental disorder. J Autism Dev Disord 22: 663–666. 1483984 10.1007/BF01046335

[10] Spiker D, Lotspeich LJ, Dimiceli S, Szatmari P, Myers RM, et al (2001) Birth order effects on nonverbal IQ scores in autism multiplex families. J Autism Dev Disord 31: 449–460. 11794410 10.1023/a:1012217807469

[11] Reichenberg A, Smith C, Schmeidler J, Silverman JM (2007) Birth order effects on autism symptom domains. Psychiatry Res 150: 199–204. 17289158 10.1016/j.psychres.2004.09.012

[12] Goin-Kochel RP, Mazefsky CA, Riley BP (2008) Level of functioning in autism spectrum disorders: phenotypic congruence among affected siblings. J Autism Dev Disord 38: 1019–1027. 17968643 10.1007/s10803-007-0476-z PMC4104536

[13] Lord C, Rutter M, Le Couteur A (1994) Autism Diagnostic Interview-Revised: a revised version of a diagnostic interview for caregivers of individuals with possible pervasive developmental disorders. J Autism Dev Disord 24: 659–685. 7814313 10.1007/BF02172145

[14] Lord C, Risi S, Lambrecht L, Cook EH Jr, Leventhal BL, et al (2000) The autism diagnostic observation schedule-generic: a standard measure of social and communication deficits associated with the spectrum of autism. J Autism Dev Disord 30: 205–223. 11055457

[15] Dunn LMaD, L M. (1997) Peabody Picture Vocabulary Tests. Circle Pines: American Guidance Service.

[16] Raven JC (1947, 1995) Colored progressive matrices: Sets I & II. Oxford: Oxford Psychologists Press, Ltd.

[17] Sparrow SS, Cicchetti DV (1984) The behavior inventory for rating development (BIRD): assessments of reliability and factorial validity. Appl Res Ment Retard 5: 219–231. 6465882 10.1016/s0270-3092(84)80003-x

[18] Bodfish JW, Symons FJ, Parker DE, Lewis MH (2000) Varieties of repetitive behavior in autism: comparisons to mental retardation. J Autism Dev Disord 30: 237–243. 11055459 10.1023/a:1005596502855

[19] Constantino JN, Davis SA, Todd RD, Schindler MK, Gross MM, et al (2003) Validation of a brief quantitative measure of autistic traits: comparison of the social responsiveness scale with the autism diagnostic interview-revised. J Autism Dev Disord 33: 427–433. 12959421 10.1023/a:1025014929212

[20] Bailey A, Phillips W, Rutter M (1996) Autism: towards an integration of clinical, genetic, neuropsychological, and neurobiological perspectives. J Child Psychol Psychiatry 37: 89–126. 8655659 10.1111/j.1469-7610.1996.tb01381.x

[21] Filipek PA, Accardo PJ, Baranek GT, Cook EH Jr, Dawson G, et al (1999) The screening and diagnosis of autistic spectrum disorders. J Autism Dev Disord 29: 439–484. 10638459 10.1023/a:1021943802493

[22] Gillberg C, Coleman M (2000) The biology of the autistic syndromes. London New York: Mac Keith Press; Distributed by Cambridge University Press. x, 330 p. p.

[23] Mottron L (2004) Matching strategies in cognitive research with individuals with high-functioning autism: current practices, instrument biases, and recommendations. J Autism Dev Disord 34: 19–27. 15098953 10.1023/b:jadd.0000018070.88380.83

[24] Lam KS, Aman MG (2007) The Repetitive Behavior Scale-Revised: independent validation in individuals with autism spectrum disorders. J Autism Dev Disord 37: 855–866. 17048092 10.1007/s10803-006-0213-z

[25] Constantino JN, Todd RD (2000) Genetic structure of reciprocal social behavior. Am J Psychiatry 157: 2043–2045. 11097975 10.1176/appi.ajp.157.12.2043

[26] Constantino JN, Todd RD (2005) Intergenerational transmission of subthreshold autistic traits in the general population. Biol Psychiatry 57: 655–660. 15780853 10.1016/j.biopsych.2004.12.014

[27] Banach R, Thompson A, Szatmari P, Goldberg J, Tuff L, et al (2009) Brief Report: Relationship between non-verbal IQ and gender in autism. J Autism Dev Disord 39: 188–193. 18594959 10.1007/s10803-008-0612-4

[28] Lord C, Schopler E (1985) Differences in sex ratios in autism as a function of measured intelligence. J Autism Dev Disord 15: 185–193. 3997745 10.1007/BF01531604

[29] Volkmar FR, Szatmari P, Sparrow SS (1993) Sex differences in pervasive developmental disorders. J Autism Dev Disord 23: 579–591. 8106301 10.1007/BF01046103

[30] Cheslack-Postava K, Liu K, Bearman PS (2011) Closely spaced pregnancies are associated with increased odds of autism in California sibling births. Pediatrics 127: 246–253. 21220394 10.1542/peds.2010-2371 PMC3387860

[31] Turner T, Pihur V, Chakravarti A (2011) Quantifying and modeling birth order effects in autism. PLoS One 6: e26418. 22039484 10.1371/journal.pone.0026418 PMC3198479

[32] Sandin S, Hultman CM, Kolevzon A, Gross R, MacCabe JH, et al.. (2012) Advancing maternal age is associated with increasing risk for autism: a review and meta-analysis. J Am Acad Child Adolesc Psychiatry 51: 477–486 e471. 10.1016/j.jaac.2012.02.018 22525954

[33] Parner ET, Baron-Cohen S, Lauritsen MB, Jorgensen M, Schieve LA, et al (2012) Parental age and autism spectrum disorders. Ann Epidemiol 22: 143–150. 22277122 10.1016/j.annepidem.2011.12.006 PMC4562461

[34] Modahl C, Fein D, Waterhouse L, Newton N (1992) Does oxytocin deficiency mediate social deficits in autism? J Autism Dev Disord 22: 449–451. 1400106 10.1007/BF01048246

[35] Insel TR, O'Brien DJ, Leckman JF (1999) Oxytocin, vasopressin, and autism: is there a connection? Biol Psychiatry 45: 145–157. 9951561 10.1016/s0006-3223(98)00142-5

[36] Modahl C, Green L, Fein D, Morris M, Waterhouse L, et al (1998) Plasma oxytocin levels in autistic children. Biol Psychiatry 43: 270–277. 9513736 10.1016/s0006-3223(97)00439-3

[37] McCauley JL, Li C, Jiang L, Olson LM, Crockett G, et al (2005) Genome-wide and Ordered-Subset linkage analyses provide support for autism loci on 17q and 19p with evidence of phenotypic and interlocus genetic correlates. BMC Med Genet 6: 1. 15647115 10.1186/1471-2350-6-1 PMC546213

[38] Lauritsen MB, Als TD, Dahl HA, Flint TJ, Wang AG, et al (2006) A genome-wide search for alleles and haplotypes associated with autism and related pervasive developmental disorders on the Faroe Islands. Mol Psychiatry 11: 37–46. 16205737 10.1038/sj.mp.4001754

[39] Ylisaukko-oja T, Alarcon M, Cantor RM, Auranen M, Vanhala R, et al (2006) Search for autism loci by combined analysis of Autism Genetic Resource Exchange and Finnish families. Ann Neurol 59: 145–155. 16288458 10.1002/ana.20722

[40] Wu S, Jia M, Ruan Y, Liu J, Guo Y, et al (2005) Positive association of the oxytocin receptor gene (OXTR) with autism in the Chinese Han population. Biol Psychiatry 58: 74–77. 15992526 10.1016/j.biopsych.2005.03.013

[41] Jacob S, Brune CW, Carter CS, Leventhal BL, Lord C, et al (2007) Association of the oxytocin receptor gene (OXTR) in Caucasian children and adolescents with autism. Neurosci Lett 417: 6–9. 17383819 10.1016/j.neulet.2007.02.001 PMC2705963

[42] Lerer E, Levi S, Salomon S, Darvasi A, Yirmiya N, et al (2008) Association between the oxytocin receptor (OXTR) gene and autism: relationship to Vineland Adaptive Behavior Scales and cognition. Mol Psychiatry 13: 980–988. 17893705 10.1038/sj.mp.4002087

[43] Hollander E, Phillips AT, Yeh CC (2003) Targeted treatments for symptom domains in child and adolescent autism. Lancet 362: 732–734. 12957098 10.1016/S0140-6736(03)14236-5

[44] Hollander E, Bartz J, Chaplin W, Phillips A, Sumner J, et al (2007) Oxytocin increases retention of social cognition in autism. Biol Psychiatry 61: 498–503. 16904652 10.1016/j.biopsych.2006.05.030

[45] Bartz JA, Hollander E (2008) Oxytocin and experimental therapeutics in autism spectrum disorders. Prog Brain Res 170: 451–462. 18655901 10.1016/S0079-6123(08)00435-4

[46] Andari E, Duhamel JR, Zalla T, Herbrecht E, Leboyer M, et al (2010) Promoting social behavior with oxytocin in high-functioning autism spectrum disorders. Proc Natl Acad Sci U S A 107: 4389–4394. 20160081 10.1073/pnas.0910249107 PMC2840168

[47] Gregory SG, Connelly JJ, Towers AJ, Johnson J, Biscocho D, et al (2009) Genomic and epigenetic evidence for oxytocin receptor deficiency in autism. BMC Med 7: 62. 19845972 10.1186/1741-7015-7-62 PMC2774338

[48] Kucharski R, Maleszka J, Foret S, Maleszka R (2008) Nutritional control of reproductive status in honeybees via DNA methylation. Science 319: 1827–1830. 18339900 10.1126/science.1153069

[49] Wolff GL, Kodell RL, Moore SR, Cooney CA (1998) Maternal epigenetics and methyl supplements affect agouti gene expression in Avy/a mice. FASEB J 12: 949–957. 9707167

[50] Dolinoy DC, Huang D, Jirtle RL (2007) Maternal nutrient supplementation counteracts bisphenol A-induced DNA hypomethylation in early development. Proc Natl Acad Sci U S A 104: 13056–13061. 17670942 10.1073/pnas.0703739104 PMC1941790

[51] Carone BR, Fauquier L, Habib N, Shea JM, Hart CE, et al (2010) Paternally induced transgenerational environmental reprogramming of metabolic gene expression in mammals. Cell 143: 1084–1096. 21183072 10.1016/j.cell.2010.12.008 PMC3039484

[52] Ng SF, Lin RC, Laybutt DR, Barres R, Owens JA, et al (2010) Chronic high-fat diet in fathers programs beta-cell dysfunction in female rat offspring. Nature 467: 963–966. 20962845 10.1038/nature09491

[53] Weaver IC, Cervoni N, Champagne FA, D'Alessio AC, Sharma S, et al (2004) Epigenetic programming by maternal behavior. Nat Neurosci 7: 847–854. 15220929 10.1038/nn1276

[54] Goines P, Van de Water J (2010) The immune system's role in the biology of autism. Curr Opin Neurol 23: 111–117. 20160651 10.1097/WCO.0b013e3283373514 PMC2898160

[55] Warren RP, Cole P, Odell JD, Pingree CB, Warren WL, et al (1990) Detection of maternal antibodies in infantile autism. J Am Acad Child Adolesc Psychiatry 29: 873–877. 2273013 10.1097/00004583-199011000-00005

[56] Zimmerman AW, Connors SL, Matteson KJ, Lee LC, Singer HS, et al (2007) Maternal antibrain antibodies in autism. Brain Behav Immun 21: 351–357. 17029701 10.1016/j.bbi.2006.08.005

[57] Braunschweig D, Ashwood P, Krakowiak P, Hertz-Picciotto I, Hansen R, et al (2008) Autism: maternally derived antibodies specific for fetal brain proteins. Neurotoxicology 29: 226–231. 18078998 10.1016/j.neuro.2007.10.010 PMC2305723

[58] Croen LA, Braunschweig D, Haapanen L, Yoshida CK, Fireman B, et al (2008) Maternal mid-pregnancy autoantibodies to fetal brain protein: the early markers for autism study. Biol Psychiatry 64: 583–588. 18571628 10.1016/j.biopsych.2008.05.006 PMC2574992

[59] Singer HS, Morris CM, Gause CD, Gillin PK, Crawford S, et al (2008) Antibodies against fetal brain in sera of mothers with autistic children. J Neuroimmunol 194: 165–172. 18093664 10.1016/j.jneuroim.2007.11.004

[60] Dalton P, Deacon R, Blamire A, Pike M, McKinlay I, et al (2003) Maternal neuronal antibodies associated with autism and a language disorder. Ann Neurol 53: 533–537. 12666123 10.1002/ana.10557

[61] Martin LA, Ashwood P, Braunschweig D, Cabanlit M, Van de Water J, et al (2008) Stereotypies and hyperactivity in rhesus monkeys exposed to IgG from mothers of children with autism. Brain Behav Immun 22: 806–816. 18262386 10.1016/j.bbi.2007.12.007 PMC3779644

[62] Singer HS, Morris C, Gause C, Pollard M, Zimmerman AW, et al (2009) Prenatal exposure to antibodies from mothers of children with autism produces neurobehavioral alterations: A pregnant dam mouse model. J Neuroimmunol 211: 39–48. 19362378 10.1016/j.jneuroim.2009.03.011

[63] Altus WD (1972) Birth order and its sequelae. In: Bronfenbrenner U, editor. Influences On Human Development. Hinsdale, Illinois: Dryden Press Inc. 600–611.

[64] El Nokali NE, Bachman HJ, Votruba-Drzal E (2010) Parent Involvement and Children’s Academic and Social Development in Elementary School. Child Development 81: 988–1005. 20573118 10.1111/j.1467-8624.2010.01447.x PMC2973328

[65] Fountain C, King MD, Bearman PS (2011) Age of diagnosis for autism: individual and community factors across 10 birth cohorts. J Epidemiol Community Health 65: 503–510. 20974836 10.1136/jech.2009.104588 PMC3039707

[66] Lovaas OI (1987) Behavioral treatment and normal educational and intellectual functioning in young autistic children. J Consult Clin Psychol 55: 3–9. 3571656 10.1037//0022-006x.55.1.3

[67] McEachin JJ, Smith T, Lovaas OI (1993) Long-term outcome for children with autism who received early intensive behavioral treatment. Am J Ment Retard 97: 359–372; discussion 373–391. 8427693

[68] Dawson G (2008) Early behavioral intervention, brain plasticity, and the prevention of autism spectrum disorder. Dev Psychopathol 20: 775–803. 18606031 10.1017/S0954579408000370

[69] Rogers SJ, Vismara LA (2008) Evidence-based comprehensive treatments for early autism. J Clin Child Adolesc Psychol 37: 8–38. 18444052 10.1080/15374410701817808 PMC2943764

